# Models of Renal Cell Carcinoma Used to Investigate Molecular Mechanisms and Develop New Therapeutics

**DOI:** 10.3389/fonc.2022.871252

**Published:** 2022-04-07

**Authors:** Daniel D. Shapiro, Maria Virumbrales-Muñoz, David J. Beebe, E. Jason Abel

**Affiliations:** ^1^Department of Urology, University of Wisconsin School of Medicine and Public Health, Madison, WI, United States; ^2^Division of Urology, William S. Middleton Memorial Veterans Hospital, Madison, WI, United States; ^3^Department of Pathology and Laboratory Medicine, University of Wisconsin School of Medicine and Public Health, Madison, WI, United States; ^4^Department of Cell and Regenerative Biology, University of Wisconsin School of Medicine and Public Health, Madison, WI, United States; ^5^Department of Biomedical Engineering, University of Wisconsin – Madison, Madison, WI, United States

**Keywords:** preclinical models, cell culture, organoid, xenograft, microfluidics, renal cell carcinoma

## Abstract

Modeling renal cell carcinoma is critical to investigating tumor biology and therapeutic mechanisms. Multiple systems have been developed to represent critical components of the tumor and its surrounding microenvironment. Prominent *in vitro* models include traditional cell cultures, 3D organoid models, and microphysiological devices. *In vivo* models consist of murine patient derived xenografts or genetically engineered mice. Each system has unique advantages as well as limitations and researchers must thoroughly understand each model to properly investigate research questions. This review addresses common model systems for renal cell carcinoma and critically evaluates their performance and ability to measure tumor characteristics.

## Introduction

Preclinical models are necessary for the discovery of molecular pathways that regulate tumor growth and to identify tumor vulnerabilities for potential therapeutic targets. Ideal models faithfully represent key characteristics of human tumors and response to drug therapies. Models should also be easily maintained and rapidly established to allow for efficient, cost-effective investigations. Additionally, models should ideally capture key components of the tumor microenvironment (TME), which is the dynamic space around the tumor, containing multiple cell types, growth and paracrine factors, and structural components (1–3). Hallmark components of the TME include immune cells, stromal cells, blood vessels and extracellular matrix (4). These components of the TME contribute to a tumor’s potential to grow or regress, metastasize, or remain localized (1–5). A deeper understanding of the TME has opened potential targets for new therapies (6, 7).

Modeling renal cell carcinoma (RCC) and the associated TME is challenging but necessary as RCC is a major cause of cancer globally. Renal cell carcinoma affects over 400,000 individuals globally per year (8). Up to one third of patients will present with or develop metastatic disease, which is almost universally fatal (9). Prognosis greatly depends on stage at diagnosis, with metastatic disease only demonstrating a 12% 5-year survival rate (8, 10). Typically, standard of care therapy for RCC consists of surgery or ablative therapy for localized disease and systemic therapy for metastatic disease (11–13). Commonly targeted pathways in RCC include the hypoxia inducible factor (HIF) and mTOR pathways, and more recently immune checkpoint blockade (14). Tyrosine kinase inhibitors and mTOR inhibitors, as well as VEGF blockade have previously been used to treat metastatic RCC until the more recent development of immune checkpoint blockade (anti-PD1, anti-PDL1 and anti-CTLA4 antibodies), which has improved disease-free and overall survival (14). Renal cell carcinoma tumors can be divided into multiple subtypes that range from indolent to aggressive and differ in their response to therapeutics. The tumors can grow large and are spatially heterogenous, consisting of multiple populations of subclones harboring unique genetic mutations in different regions of the tumor (15, 16). Additionally, RCC tumors can modulate the host immune response, which has become the target for modern systemic immune-based therapies (17). No single preclinical model can capture the complex tumor microenvironment or the subsequent host response. Thus, researchers employ multiple systems to capture and control unique components of RCC tumor biology to answer specific questions. The focus of this review will be to discuss commonly used preclinical models for RCC mechanistic and therapeutic investigations.

## Renal Cell Carcinoma Genetic Hallmarks

**Figure 1:**
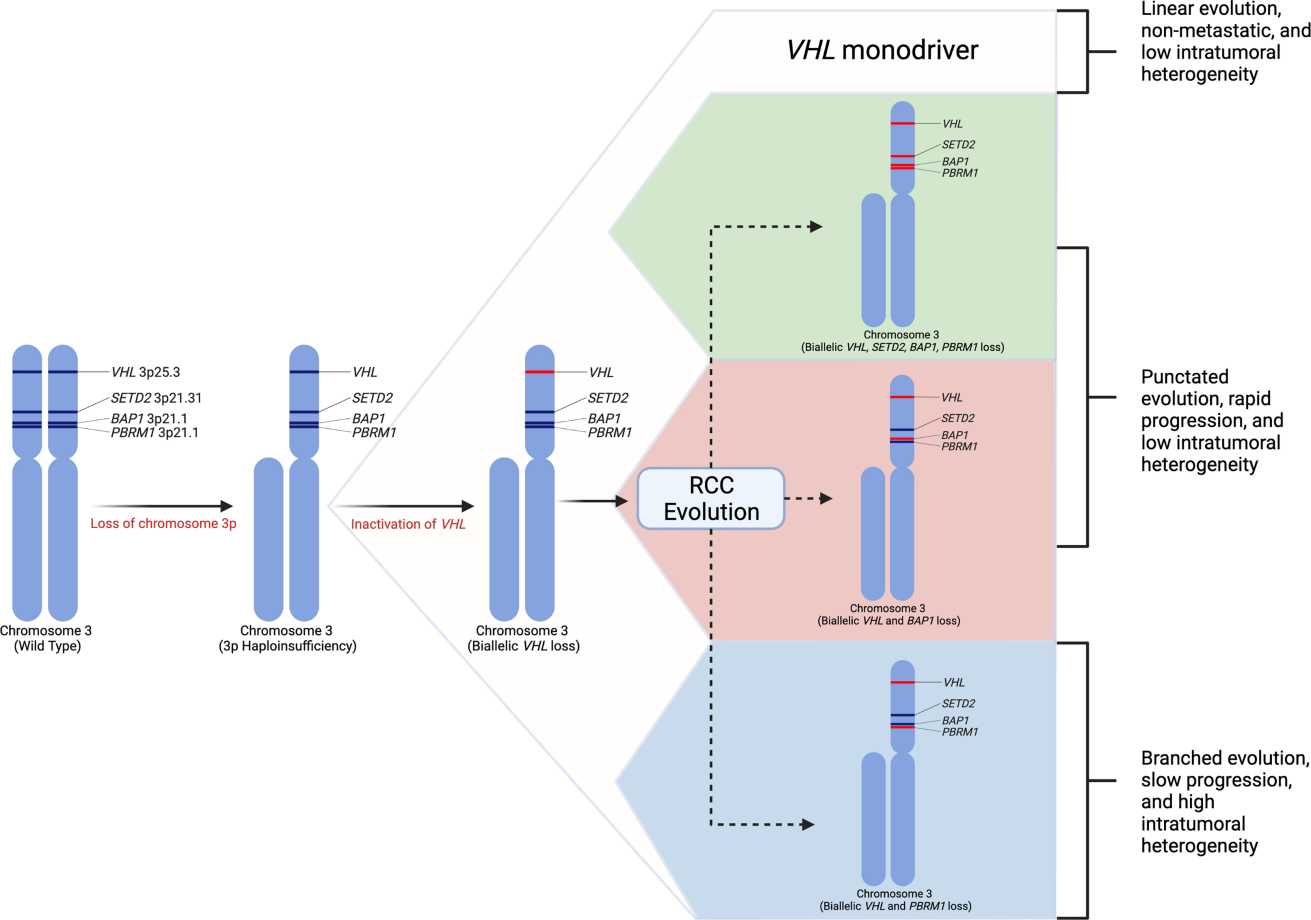
Renal cell carcinoma genetic evolution. This model of renal cell carcinoma evolution demonstrates chromosome 3p loss as the initial “first-hit” event. Subsequent biallelic loss of *VHL* occurs as a second hit. Tumor cells then may undergo multiple different pathways or “branches” of evolution based on mutations in other driver genes such as *SETD2, PBRM1*, and *BAP1*. These distinct evolutionary branches create different tumor phenotypes (31).

Selecting biologically relevant preclinical models for research requires a thorough understanding of the molecular hallmarks of RCC. Renal cell carcinoma arises from epithelial cells of the nephron. RCC is a heterogenous disease (15) and can be classified into distinct histologic subtypes of which clear cell RCC (ccRCC) is the most common, accounting for about 70-80% kidney cancers (9, 18–20). Other common subtypes include papillary type 1, papillary type 2, and chromophobe RCC; however, a number of other unique subtypes exist (21). Clear cell renal cell carcinoma is defined by loss of the *VHL* gene expression (22–24). Studies have demonstrated that the most frequent arm-level events involve loss of chromosome 3p (91% of samples) (16, 22). Loss of this allele includes loss of *VHL* as well as additional tumor suppressor genes in close proximity to *VHL* including *PBRM1, BAP1*, and *SETD2* (22, 25, 26). These four genes become the targets for subsequent inactivating mutations on the second chromosomal copy (22, 25). While second-hit loss of function mutations in *VHL* are present in over 90% of ccRCC, complete loss of *VHL* expression alone is insufficient to produce ccRCC in humans as well as mice (27, 28). The most frequently mutated genes in ccRCC besides *VHL* are *PBRM1* (~45%), *SETD2* (10-15%), and *BAP1* (10-15%) (22, 29, 30). Other common mutations found in the TCGA cohort include *MTOR, PIK3CA, KDM5C, TP53* and *PTEN* (22, 29). Using a multi-region sampling approach, the TRACERx consortium evaluated the evolutionary trajectories of ccRCC (25). This study demonstrated that loss of chromosome 3p is the initiating driver event, which typically occurs in childhood or adolescence (25). The second critical event is the inactivation of the second *VHL* allele which may occur decades after the initial 3p loss. Additional driver mutations in *PBRM1, SETD2*, and *BAP1* may occur, and the number of driver events is significantly associated with tumor stage, grade, and presence of necrosis (**Figure 1**) (16, 25). *VHL* inactivation leads to loss of VHL protein, which is a critical component of the E3 ubiquitin ligase complex that functions to ubiquitinate hypoxia-inducible transcription factors in the presence of oxygen, leading to proteasomal degradation (23). Loss of the VHL protein expression causes stabilization of the hypoxia inducible factors (HIF-1α and HIF-2α). Unimpeded HIF activation leads to expression of target genes regulating angiogenesis, glycolysis, and apoptosis (23). The next most commonly modified genes, including *PBRM1, SETD2*, and *BAP1*, are all chromatin modifiers and play roles in chromatin maintenance (32). Mutations in *BAP1* have correlated with poor survival in ccRCC and *PBRM1* mutations have been associated with a more indolent phenotype (18). Additional cross-platform molecular analyses demonstrated a correlation between worsened prognosis in patients with ccRCC harboring mutations causing metabolic shifts involving increased reliance on the pentose phosphate shunt, decreased AMPK, decreased Krebs cycle activity, increased glutamine transport and fatty acid production (33). This metabolic shift is similar to the Warburg metabolic phenotype of increased glycolysis and decreased AMPK, glutamine-dependent lipogenesis (22). In general, primary RCC tumors can demonstrate profound intratumoral genomic heterogeneity (15), and multi-region sequencing of primary tumors have demonstrated that up to seven biopsies are required to detect at least 75% of the many subclonal driver events (16).

Loss of chromosome 9p and 14q appear to be hallmark genomic alterations in ccRCC metastatic competence (31). Additionally, distinct genomic evolutionary subtypes have been identified. The order and timing of driver events leads to distinct metastatic potential. There appear to be two primary modes of metastatic dissemination. Primary tumors with decreased intratumoral heterogeneity and high genomic instability acquire metastatic ability early in tumor clonal evolution leading to rapid progression (31). Among these tumors, subtypes exist including tumors in which metastatic competence is driven by *BAP1* inactivation, tumors that maintain *VHL* wild-type expression, or tumors that contain multiple clonal drivers (31). Conversely, primary tumors with high intratumoral heterogeneity with or without high genomic instability gradually acquire metastatic capacity causing attenuated metastatic progression. These subtypes include *PBRM1* followed by *PI3K* inactivation, *PBRM1* followed by *SETD2* inactivation, and *PBRM1* followed by somatic copy number alterations (31). Genomic characterization of metastatic sites among these subtypes demonstrated more homogenous cells with fewer somatic driver alterations (31). Tumors with high intratumoral heterogeneity and low genomic instability typically demonstrate an initial oligometastatic pattern with gradually increasing metastatic load over time (31).

Beyond ccRCC, additional subtypes of RCC continue to be described with their own unique genetic hallmarks. Fumarate hydratase (FH) deficient RCC is characterized by mutations in the FH gene at 1q42. Patients often present with a hereditary leiomyomatosis and renal cell carcinoma (HLRCC) syndromic findings including cutaneous leiomyomas and uterine leiomyomas in females in addition to type 2 papillary RCC tumors. These tumors have reportedly responded to first-line bevacizumab/erlotinib (34). Succinate dehydrogenase (SD) deficient RCC is due to inactivation of one of the genes within the SDH family, often as germline mutations. In addition to kidney tumors, patients may develop pheochromocytomas, GIST and pituitary adenomas (34). MiT family translocation RCC (tRCC) account for around 50% of pediatric RCC and are caused by gene fusions of TFE3 or TFEB. The most common translocation involves Xp11 leading to activation of TFE3 (34). Other rare renal tumors with distinct genetic characterizations include ALK rearrangement-associated RCC (ALK-RCC), TCEB1 renal cell carcinoma, renal medullary carcinoma, collecting duct carcinoma, tubulocystic RCC, clear cell papillary RCC, acquired cystic disease-associated RCC, and mucinous tubular spindle cell carcinoma (34).

Overall, the understanding of these molecular mechanisms has come from work with models of renal cell carcinoma including cell lines, organoids, and murine models. These models can help to recapitulate the molecular mechanisms of human RCC. We will provide a review of the main preclinical models utilized when studying renal cell carcinoma.

## Renal Cell Carcinoma Cell Lines

Cell lines provide a low-cost research tool to understand the molecular mechanisms of RCC. Ideal cell lines will faithfully recapitulate defining tumor characteristics such as the critical genomic changes that drive RCC tumor phenotypes (35). Additionally, ideal cell lines will respond to new therapies (e.g., new drug therapies) in a way that predicts outcomes in humans. The first established cell lines for RCC included 786-O, ACHN, CAKI-1, A498, RXF393, UO-31, TK-10, and SN12C (36). These were established as part of the US National Cancer Institute 60 cell line anticancer drug screen effort (36, 37). There are a substantial number of additional RCC cell lines that are derived from various RCC subtypes including lines derived from both primary renal tumors as well as metastatic sites (38). In addition to the commonly used and commercially available immortalized cell lines, more recent efforts have been made to create individual patient derived cell lines that maintain the characteristics of the parental tumor, including parental driver mutations and allele frequency (39). These cell lines may allow for development of personalized drug screening and discovery (39). Each cell line harbors a unique genetic profile which is critical to understand prior to interpreting genetic and cellular events during *in vitro* studies (**Table 1**) (38). We will review some of the most frequently used cell lines in RCC research.

**Table 1:** Common renal cell carcinoma cell lines (36, 40, 41).

Cell Line	RCC Subtype	Phenotype	*VHL*	Mutations in key RCC genes per CCLE (41)
786-O	Clear cell	Sarcomatoid features	Null (p.G104fs)	*PTEN* (p.Q149*) *TP53* (p.R248W)
ACHN	Papillary	Sarcomatoid features	Wildtype	*PBRM1* (p.R1067*) *NF2* (p.R57*)
A-498	Clear cell	Clear cytoplasm	Null (p.VD142fs)	*SETD2* (p.V2536fs) *MLL3* (p.G2986D)
CAKI-1	Clear cell	Poorly differentiated	Wildtype	*SETD2* (p.)? *MET* (p.V1238I)
CAKI-2	Clear cell	Well-differentiated	Null (p.R177*)	*PBRM1* (p.IY876fs)
769-P	Clear cell	Not reported	Null (p.I180N)	*BAP1* (p.Y33D) *TSC2* (p.I606V)
UMRC-2*	Clear cell	Not reported	Null	N/A

*Not characterized by CCLE database. RCC, renal cell carcinoma; CCLE, Cancer cell line encyclopedia. N/A, Not Available.

The ACHN cell line was derived from a malignant pleural effusion patient sample and appears to be possibly derived form a papillary RCC subtype. This cell line harbors a *MET* mutation consistent with papillary RCC (38). Additionally, an analysis of copy number alterations reveal that ACHN cells cluster with papillary tumors when compared to TCGA renal tumor copy number alterations (40). The ACHN cell line is the third most commonly cited cell line in the literature, despite being derived from papillary RCC cells (36, 40). When investigating key RCC mutations, ACHN cells were found to harbor no mutations in genes including *VHL, PBRM1, SETD2, BAP1*, and *TP53* (40). The ACHN line was used by Thomas et al. to demonstrated that after VHL knockdown, ACHN cells became sensitive to mTOR inhibition, providing the support for the clinical use of mTOR inhibitors for RCC treatment (42). Histologically, xenografts derived from ACHN cells tend to show a poorly differentiated carcinoma with aggressive features (40). Despite its genomic correlation with papillary RCC, ACHN tends to show sarcomatoid differentiation rather than papillary architecture in xenografts (40). Given its likely papillary origins, investigators should use caution if using this cell line in an attempt to recapitulate clear cell RCC diseases.

The 786-O cell line is one of the most frequently used cell lines for RCC specific research (36, 38). 786-O cells are characterized by their loss of function mutations in *VHL* genes leading to clear cell RCC phenotypes (36, 40, 43). These cells demonstrate increased expression of HIF-2α and VEGF proteins. This cell line has been used in numerous studies including initial studies demonstrating the function of the VHL/HIF pathway (24, 36, 44). In a study by Iliopoulos et al, restoration of pVHL in 786-O cells effectively suppressed tumor growth in nude mice (24). Kondo et al. demonstrated that the tumor suppression gained by pVHL can be overridden by a HIF variant that escapes pVHL control. This effectively demonstrated HIF as a target of pVHL and that unsuppressed activation of HIF promotes tumorigenesis (24, 44). Additionally, 786-O cells were used to demonstrate that inhibition of HIF-2α with shRNA suppressed tumor growth in 786-O cell derived xenograft models (44). About 2 decades later, belzutifan, a small molecule HIF-2α inhibitor would receive FDA approval for treatment of patients with VHL disease (45). While 786-O appears to have been derived from ccRCC, the cell line has undergone significant dedifferentiation *in vitro*, both genomically and histologically. It demonstrates a sarcomatoid appearance in xenograft studies and the cells do not display a clear cytoplasm characteristic of typical ccRCC histology in humans (36, 40).

Another frequently cited cell line is A-498. This cell line contains p.VD142fs frameshift deletion of *VHL*, suggesting its ccRCC origin (36, 41). This cell line has been classified according to the prognostic expression based subtype ccB, which indicates a more aggressive phenotype, along with other RCC cell lines 786-O, CAKI-1, and OS-RC.2 (40). RCC tumors have previously been classified according to the ccA (“clear cell A”) and ccB prognostic classification system, which are defined according to a highly predictive gene set developed using 48 RCC tumors and subsequently validated using 177 RCC tumors (46). ccA tumors display increased angiogenesis and hypoxic signaling while ccB tumors display decreased hypoxic gene expression, elevated epithelial-to-mesenchymal signaling and more aggressive behavior (46). While both 786-O and A-498 appear to derive from a ccRCC origin, 786-O tends to harbor a greater number of genetic alterations than A-498. Additionally, while 786-O demonstrates poorly differentiated cells histologically, A-498 cell derived xenografts demonstrate more typical histologic findings of ccRCC including nests of malignant cells with clear cytoplasm (40). The A-498 cell line has been used to demonstrate the upregulation of the mTOR signaling pathway in RCC and demonstrated that cell growth could be inhibited by the mTOR inhibitor, rapamycin (47).

Overall, multiple different cell lines derived from RCC tumors exist from which researchers can choose. These cell lines contain a diverse genetic makeup and display variable phenotypes *in vitro* and when engrafted *in vivo* to make xenografts. As noted in **Table 1**, *VHL* is wild-type in 3 of the 6 most commonly reported cell lines, yet *VHL* is known to be mutated in over 90% of ccRCC tumors found in patients. This creates a divergent molecular phenotype among cell lines compared to tumors found in the majority of patients. Some studies have investigated generation of ccRCC patient derived cell lines to search for therapeutic vulnerabilities at an individual level (48). This can be challenging as patient derived cell lines are frequently contaminated by normal epithelial cells leading to poor efficiency (48). It is critical for the researcher to select the appropriate cell line based on the research question being investigated and select one that most closely resembles the tumor molecular response and phenotype *in vivo*. Cell cultures have other significant limitations. Traditional cell cultures grow in only 2 dimensions compared to the 3-dimensional tumor architecture. Cell lines typically become de-differentiated and pick up mutations after serial passages *in vitro*.

## Organoid Models

New model techniques have been developed to overcome some of the limitations found using traditional cell cultures. It is known that the structural phenotype of the tumor influences cellular interactions and subsequent gene expression (35, 49). Thus, 2D cell cultures are limited by their lack of normal tissue architecture. Organoids are 3D cell cultures that attempt to mimic normal and tumor tissue structure *in vitro* (50, 51). These 3D models imitate environmental pressures in culture such as nutrient gradients in which the inner portion of the tumors receive less nutrients due to devascularization, and also more realistically model how drug therapies reach different portions of the tumors (51, 52). These qualities allow researchers to study tumor drug-sensitivity in addition to other mechanistic and genomic studies (51). Organoid cultures have been utilized in many cancers including breast (53), prostate (54), pancreas (55), colorectal cancer (56), as well as in RCC.

A study by Grassi et al. established normal kidney and renal cell carcinoma organoids. Using portions of excised RCC tumors, the tissue was homogenized both mechanically and enzymatically. The cell suspension was first plated in a stem cell enriching medium and after an incubation period, the cells were transferred to Matrigel containing organoid specific medium to promote organoid growth (50). Tumor organoid colonies were noted to establish at a lower rate than benign kidney derived organoids. Additionally, tumor organoids notably lost their structure after serial passages (50). Sequencing studies demonstrated preserved mutational patterns among organoids when compared to the primary tumor sample, indicating organoids may make excellent patient specific models for personalized drug susceptibility testing (50). It should be noted that Matrigel is frequently used for organoid culture but is a poorly characterized medium derived from mouse sarcoma cells and may alter normal cellular function (57). New methods are being developed to grow organoids without Matrigel (57).

Organoids also provide a platform for incorporating other components of the tumor microenvironment beyond sole tumor cells. Renal cell carcinoma has seen recent treatment advances based on immune activating therapies (58–66). One difficulty in the modern era of immunotherapy for advanced clear cell RCC is developing a model system that incorporates both tumor cells and the surrounding tumor immune microenvironment (TME). These models are critical to discovering potential biomarkers to guide immunotherapy (66, 67). To recapitulate the TME, Neal et al. developed air-liquid interface organoids which preserve primary tumor epithelium en bloc with endogenous immune and non-immune stromal components (68). This organoid model allows for the study of immunotherapy effects *in vitro*, and this model demonstrated a functional tumor infiltrating lymphocyte response to PD-1/PD-L1 checkpoint blockade (68). While these models better incorporate components of the TME, they cannot measure the effects of the peripheral immune system, which are also critical for anti-tumor immunity (68). Using the same air-liquid interface system, Esser et al. generated patient derived organoids from 42 RCC tumors (69), which better represented the primary tumors as demonstrated by IHC and RNA sequencing analysis (69). Additionally, they used 10 organoids and treated them with either cabozantinib or nivolumab, and they noted different responses of each organoid to either of these therapies. Response to nivolumab was dependent on a higher CD8+ T-cell presence in the organoid model (69). The authors demonstrate that patient derived organoids appear to be a suitable tool for therapeutic testing even with immunotherapeutic agents (69).

Overall, organoids provide advantages over traditional 2D cell cultures in that they more accurately reflect the 3-dimensional tissue architecture and even can mimic properties of the TME including nutrient gradients and incorporating immune or stromal cells. They are, however, more labor and time intensive, and can be limited by higher costs than 2-dimensional cell lines. Additionally, similar to 2D cultures, organoids can develop genetic alteration differences from the primary tumor as the organoids are serially passaged and subjected to *in vitro* selection pressures (70). Regardless, these preclinical models provide a rational design for testing personalized targeted therapeutic regimens and warrant further prospective investigations.

## Microfluidic (Microphysiological) Models

**Figure 2:**
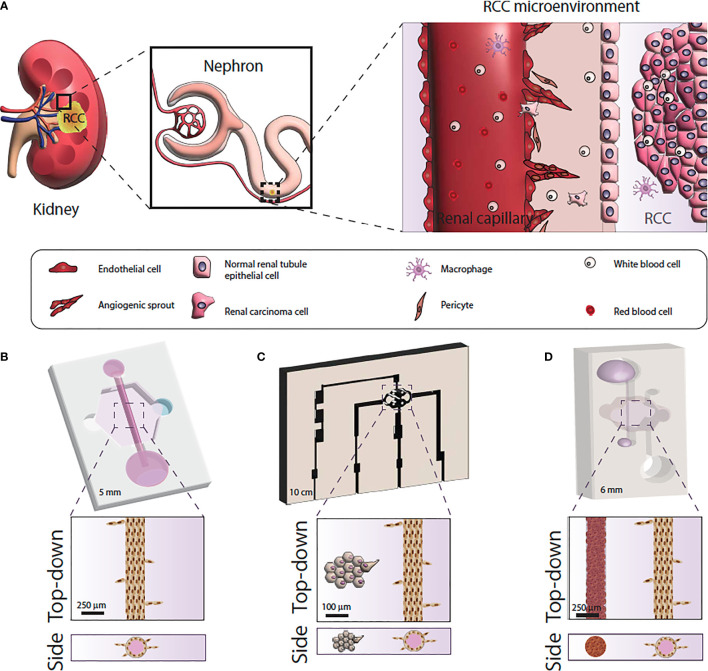
Microfluidic model systems used for renal cell carcinoma research. **(A)** Schematic of the RCC tumor microenvironment. **(B)** Microfluidic model of RCC response using primary normal and tumor-associated endothelial vessels. **(C)** Microfluidic model of RCC angiogenesis using primary epithelial-derived spheroids and Human Umbilical Cord Endothelial cell vessel models. **(D)** Microfluidic of RCC development and angiogenesis using RCC cell lines or primary epithelial cells and iPSC-derived endothelial cell vessels.

The last few years have seen an explosion in microfluidic tumor models, often called microphysiological models (71). Microfluidic models are housed in microscale *in vitro* devices that allow for the manipulation of fluids at microliter volumes to control microenvironmental conditions, create biochemical gradients and allow for work with extremely small quantities of samples (72, 73). Of note, microfluidic devices are especially useful for recreating different compartments and geometries to generate physiologically-relevant structures and generate organized co-cultures for *in vitro* studies (**Figure 2**).

Although the field of microfluidics started to develop in the early 2000s, only recently have microfluidic models come of age and gained acceptance as *in vitro* tools (74). Therefore, microfluidic models of normal kidney function (or kidney-on-a-chip) have been reported since the early 2010s (75). These models initially consisted of 2D cultures of kidney cell lines where fluid flow was applied to observe the effects of this mechanical stimulus on kidney cell behavior (e.g., changes in morphology, inflammation) (76). However, overtime these models have evolved to include biologically derived matrices, relevant tubular geometries to recreate the renal tubule, and a nearby blood vessel model to mimic a nephron more closely (77). To date, these studies used models to investigate normal kidney function and nephrotoxicity (78–80).

However, microfluidic models are particularly relevant in RCC research. Renal cell carcinoma produces significant angiogenic signals leading to neovascularity formation. Tumor-associated vessels differ from normal vessel in organization, angiogenic sprouting and vessel permeability (81). Traditional *in vitro* models have been unable to recapitulate tumor-induced angiogenesis, but microfluidics have demonstrated promise in modeling tumor neovascularity (82) and been used to test drugs that target angiogenesis (81).

Despite the great potential of microfluidic modeling in RCC research, few microfluidic RCC models exist. Researchers have mimicked the 3-dimensional vascular microenvironment of RCC by placing tumor cell clusters around engineered human vessels that are subjected to continuous flow (83). Using this design, one study demonstrated that ccRCC cells could stimulate angiogenesis due to the tumor cell upregulation of angiogenic factors, and create what the investigators termed “ccRCC-on-a-chip” (83). Another study used patient-derived normal and tumor-associated endothelial cells to create microfluidic tubular vessel models from multiple patients (81). The microfluidic device demonstrated higher permeability in vessel models built from tumor-associated endothelial cells, recapitulating the phenotypic differences between normal and tumor associated microvasculature. The researchers were able to test anti-angiogenic drugs using the microfluidic devices which caused decreased vessel sprouting and restored tumor vessel permeability (81). A recent study used cell lines and induced pluripotent stem cell-derived endothelial cells to recapitulate RCC development and angiogenic response. Specifically, the authors recapitulated RCC hallmarks like hypoxia, glycolic metabolism, and sprouting angiogenesis, and then tested the model response to a common anti-angiogenic drug. The authors also demonstrated the same model using patient-derived RCC cells instead of cell lines with equivalent results (84).

Microfluidic modeling presents multiple advantages for *in vitro* research, such as inclusion of many cell types, integration of mechanical cues (e.g., extracellular matrices, perfusion of media) and real-time monitoring capabilities. The high degree of control in microfluidic devices makes these models ideally suited for anti-cancer drug testing studies and investigations into mechanisms of pathology. These types of physiological investigations are more difficult using *in vivo* models in which the tumor cannot be easily separated into certain components (such as only investigating the tumor cell interaction with the surrounding microvasculature) (72). Existing models are limited by only incorporating 1-2 cell types, thereby failing to include the complex interactions between multiple cell types such as tumor, stromal, and immune cells in RCC (85, 86). Thus, existing RCC research using these devices has typically focused on a single physiologic process within the tumor environment, but there is great potential for more complex RCC modeling using microfluidics (72, 87).

## Xenograft Models of Renal Cell Carcinoma

While 2- and 3-D cultures provide an efficient, low-cost way to study cellular mechanisms and potential responses to drug therapies, these models are ultimately limited by their inability to capture the heterogenous tumor microenvironment *in vivo* including tumor interactions with the surrounding stromal and inflammatory cells. One method to overcome this limitation is to utilize mouse models bearing RCC tumors. These tumors can be generated from a variety of sources. One method involves the xenotransplantation of established RCC cell lines (also known as cell derived xenografts). Another option includes transplantation of tumor tissue taken directly from patient tumor samples into immunodeficient mice to allow growth of the tumors. These tumors are known as “patient derived xenograft” models or PDX models. Newer advances in genetic engineering have allowed for the creation of genetically engineered mouse models (GEMMs), which are discussed below. GEMMs are advantageous because they can mimic the genetic composition, cellular interactions, and therapeutic response of human tumors (88). Cancer cell line transplantation into mice is a commonly used method allowing for rapid preclinical drug testing (88), however these models often poorly predict clinical response in humans. PDX models more faithfully recapitulate the genetic intratumoral heterogeneity and can allow for a personalized medicine approach to drug screening (88, 89). These tumor models are limited by low engraftment rates and must be performed in an immunocompromised mouse, making immunotherapy testing impossible. This is a significant limitation in the current immunotherapy dominant era for advanced RCC treatment (88–90).

Multiple studies have demonstrated the feasibility of developing patient derived xenograft models of RCC. Grisanzio et al. developed a panel of PDX models in immunodeficient mice (91). The authors implanted 20 patient-derived RCC tumors orthotopically (under the renal capsule) using clear cell, papillary and oncocytoma tumors. They demonstrated a high engraftment rate at 95% and found PDX tumors maintained the same genetic characteristics of the original tumors (91). They also noted two cases that developed locally invasive disease and one that developed distant metastases (91). Sivanand et al. established PDX models to investigate molecularly targeted therapies (92). They found engraftment rates were higher for tumors derived from metastatic sites compared to primary kidney tumors (80% versus 14%, respectively) (92). Additional studies have supported this finding, demonstrating higher engraftment rates derived from metastatic sites compared to primary tumors (93). In a large-scale study of PDX model development, Lang et al. implanted 336 RCC tumors of various histologic subtypes into nude mice, with a stable engraftment rate (i.e., greater than 3 successful passages) of 8.9% (94). The authors found higher stable engraftment rates with increasing stage and grade of primary tumors, as well as among tumors with sarcomatoid features. Importantly, the PDX models maintained their genetic characteristics after serial passages. The models also showed significant variation after exposure to different targeted therapies including sunitinib, sorafenib and everolimus, similar to tumor responses in humans (94). Stable engraftment of PDX tumors has shown prognostic value and been associated with worse survival in humans, suggesting a more aggressive tumor phenotype when tumors are able to stably engraft in mice (92, 95, 96). Sivanand et al. additionally evaluated PDX tumor response to targeted therapies sunitinib and sirolimus. Both therapies inhibited PDX tumor growth similar to responses in human tumors (92). They further tested the investigational drug dovitinib, which inhibits VEGFR1-3, PDGFRβ, and FGFR1-3. Dovitinib inhibited PDX tumor growth to a greater extent than either sunitinib or sirolimus, and was well tolerated by the mice (92). This study demonstrated the applicability of using PDX models as a model of human tumor clinical outcomes and responses to targeted therapies, which do not require an active immune system to work. Dong et al. importantly demonstrated that PDX models more accurately represented responses to sunitinib than cell lines derived from the same primary tumor, suggesting that the *in vivo* models of RCC more faithfully recapitulate human tumor responses than the patient derived cell lines (93).

More recent developments in PDX models include creating a “humanized” mouse model. Humanized mouse models are created by transplantation of a human tumor graft into an immunodeficient mouse and simulating the human immune system by simultaneously transplanting human peripheral blood or tumor infiltrating lymphocytes into the mouse model. This is advantageous because it allows for testing immunotherapeutics within PDX models, which traditionally lack components of the immune system (97). Another method for generating humanized models involves the transplantation of human hematopoietic stem cells (HSCs), allowing for more complete restoration of all hematopoietic cells. These models are limited by the availability of HSCs able to be obtained from cancer patients (98). While humanized PDX models allow for better evaluation of immune therapies, they still are limited by the lack of incorporation of all cell types, robust or reliable immune response, and the frequent development of graft versus host disease in the mouse (98). PDX models have been used as a robust platform for rapid preclinical drug testing and many centers continue to maintain large tumorgraft colonies derived from patient tumor samples (99, 100). These will continue to be widely used as preclinical models for RCC-based studies.

## Genetically Engineered Mouse Models

Genetically engineered mouse models (GEMMs) provide a method to study tumorigenesis and metastasis as well as therapeutic testing similar to PDX models. GEMMs are particularly attractive for oncology research due to the relative ease the mouse genome can be manipulated to inactivate tumor suppressors or activate oncogenes (101, 102). These models can be used in a variety of ways for translational research. GEMMs can be used to test novel drug targets, evaluate therapeutic response as well as mechanisms of resistance, and may be used for immunotherapeutic research as these models typically maintain an intact immune system (88). Current GEMMs allow for the conditional somatic inactivation of tumor suppressor genes or activation of oncogenes allowing for tissue specific tumorigenesis (88, 103). Mechanisms to achieve this often utilize Cre-*loxP* system. Cre-recombinase is a DNA recombinase derived from bacteriophage that causes recombination at 34 base-pair recognition sites called *loxP* (104). Genes flanked by the *loxP* recombination site will be deleted after Cre-recombinase activation is achieved such as through cellular delivery of Cre-recombinase by adenovirus (88). Somatic mutations can also be induced at specific times by utilizing Cre-ERT fusion proteins (88). The estrogen receptor binding domain is fused to Cre-recombinase, and administration of tamoxifen to the mouse causes the expression of Cre-recombinase leading to excision of *loxP* flanked genes (88). These systems allow for immunocompetent mouse models which produce *de novo* tumors that mimic human tumors.

**Figure 3:**
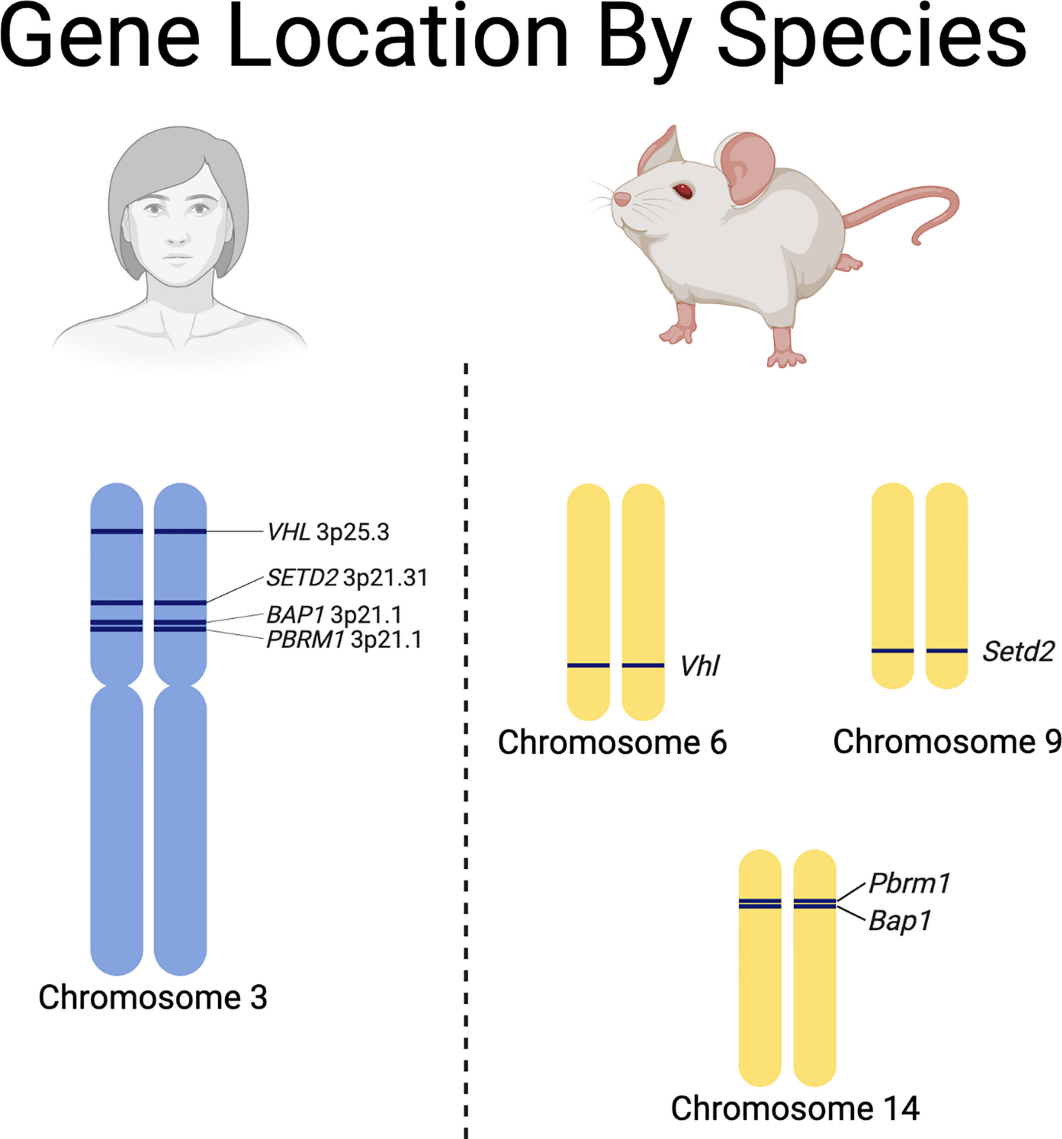
Species specific differences in the chromosomal locations of renal cell carcinoma driver genes.

While valuable, they can be labor intensive, expensive, slow to develop tumors, and require a thorough understanding of the differences between some of the RCC driver gene locations in humans and mice (**Figure 3**) (88). Recent advances have led to the development of autochthonous RCC models (27, 105–109). Initial efforts to generate GEMMs of ccRCC focused on inactivation of *Vhl* in mice, since biallelic inactivation of *VHL* is present in over 90% of sporadic ccRCC in humans (28, 110–112). Interestingly, multiple groups attempted to generate ccRCC tumors in mice through either embryonic or conditional knockout of *Vhl* in mice without successful development of tumors (105, 108). This led to the realization that additional mutations are necessary to generate ccRCC tumors in humans and mice (105, 108). In humans, the *VHL* gene is located next to other tumor suppressors (e.g., *PBRM1, BAP1* and *SETD2*) on chromosome 3p. One explanation for the lack of development of ccRCC in mice by sole knockdown of *Vhl* is that the mouse *Vhl* gene is located on chromosome 6 while *Pbrm1* and *Bap1* are located on chromosome 14 and *Setd2* is located on chromosome 9 (**Figure 3**). Thus, loss of *Vhl* in mice still leaves functional *Pbrm1, Bap1*, and *Setd2* genes, while loss of chromosome 3p, which is the typical first-hit event in humans, often causes loss of *PBRM1, BAP1 and, SETD2*, predisposing humans to additional second-hit inactivating mutations in these genes and subsequent tumor development (107).

Harlander et al. demonstrated that mutations in mouse *Vhl* combined with *Trp53* and *Rb1* in renal epithelial cells successfully generated ccRCC tumors in mice (27). To do this, the authors utilized the inducible renal epithelial cell specific Ksp1.3-Cre^ERT2^ homozygous deletion of the *loxP*-flanked alleles of *Rb1, Vhl*, and *Trp53*. These deletions were induced by exposing mice to tamoxifen. Eighty-two percent of the triple deletion mice developed renal tumors with histologic and transcriptional similarities to human ccRCC (27). The authors further treated mice with sunitinib and demonstrated, similar to humans, heterogenous responses to the drug. After exposure to sunitinib, 32% of tumors progressed, 16% of tumors regressed and 53% of the tumors remained stable in size (27). While *VHL, TP53, and RB1* are not common coincident mutations in ccRCC, the investigators speculate that this background mimics the copy number alterations of p53 pathway regulators and cell cycle control enzymes typically found in human ccRCC tumors (27). This model permits the evolution of genetically distinct ccRCC in each mouse (27).

Gu et al. developed a GEMM using a *Pax8-Cre* deletion of *Vhl* along with *Bap1* and *Pbrm1* (107). Deletion of *Vhl* and *Bap1* led to expected changes in effector pathways including upregulation of *Hif-1* and *Hif-2* as well as increased ubiquitinated histone H2A protein which is normally deubiquitinated by BAP1 (107). Creation of mice that were either deficient in *Bap1* or *Pbrm1* along with *Vhl* developed multiple cystic and solid lesions in the kidneys similar to human ccRCC (107). Also similar to humans, *Bap1* deficient tumors tended to be higher grade compared to *Pbrm1* deficient tumors which were slower to develop, more homogenous and lower grade (29, 107). Bailey et al. developed papillary RCC GEMMs through renal tubular cell specific activation of MYC (109). When the authors combined MYC activation with *Vhl* and *Cdkn2a* deletion, kidney tumors more closely resembled ccRCC (109). Clear cell RCC frequently contains focal gains of 8q24 which harbors the *MYC* gene, as well as losses of 9p21 which harbors *CDKN2A* (22, 109, 113). This model was unique in that it demonstrated metastatic capability, with 1/3 of the mice developing liver metastases. This model is limited in that only about 6% of human ccRCC tumors have *VHL* and *CDKN2A* inactivation with *MYC* overexpression (109).

These studies demonstrate the utility of GEMMs in recapitulating human RCC and the ability to use these models to study mechanisms of tumorigenesis (**Table 2**). These models, however, require a thorough understanding of the human and mouse genome and gene-expression differences, which often explain their phenotypic differences between mouse and human pathology (114, 115).

**Table 2:** Genetically engineered mouse models used for kidney cancer research.

	*Vhl^-/-^ Pbrm1^-/-^* (106, 107)	*Vhl*^-/-^ *Bap1*^+/-^ (108)	*Myc* overexpression + *Vhl*^-/-^ *Cdkn2a*^-/-^ (109)	*Vhl*^-/-^ *Trp53*^-/-^ *Rb1*^-/-^ (27)
**Subtype**	ccRCC	ccRCC	ccRCC	ccRCC
**Advantages**	•100% tumor generation •Multiple tumors formed •Low grade	•High grade tumor development •Lymphovascular invasion •Increased HIF-1, HIF-2 and mTORC1	•High grade tumor development •Liver metastases generated in 1/3 of mice •Short latency period for tumor development •Increased gene expression related to epithelial mesenchymal transition	•High grade tumor development •Nuclear accumulation of HIF-1α and HIF-2α
**Limitations**	•No metastases	•No metastases •Small lesions •High mortality	•Less representative of traditional ccRCC (~6% have *VHL* and *CDKN2A* inactivation with *MYC* activation)	•No metastases •*VHL, TP53* and *RB1* inactivation not common in human ccRCC

## Conclusion

**Figure 4:**
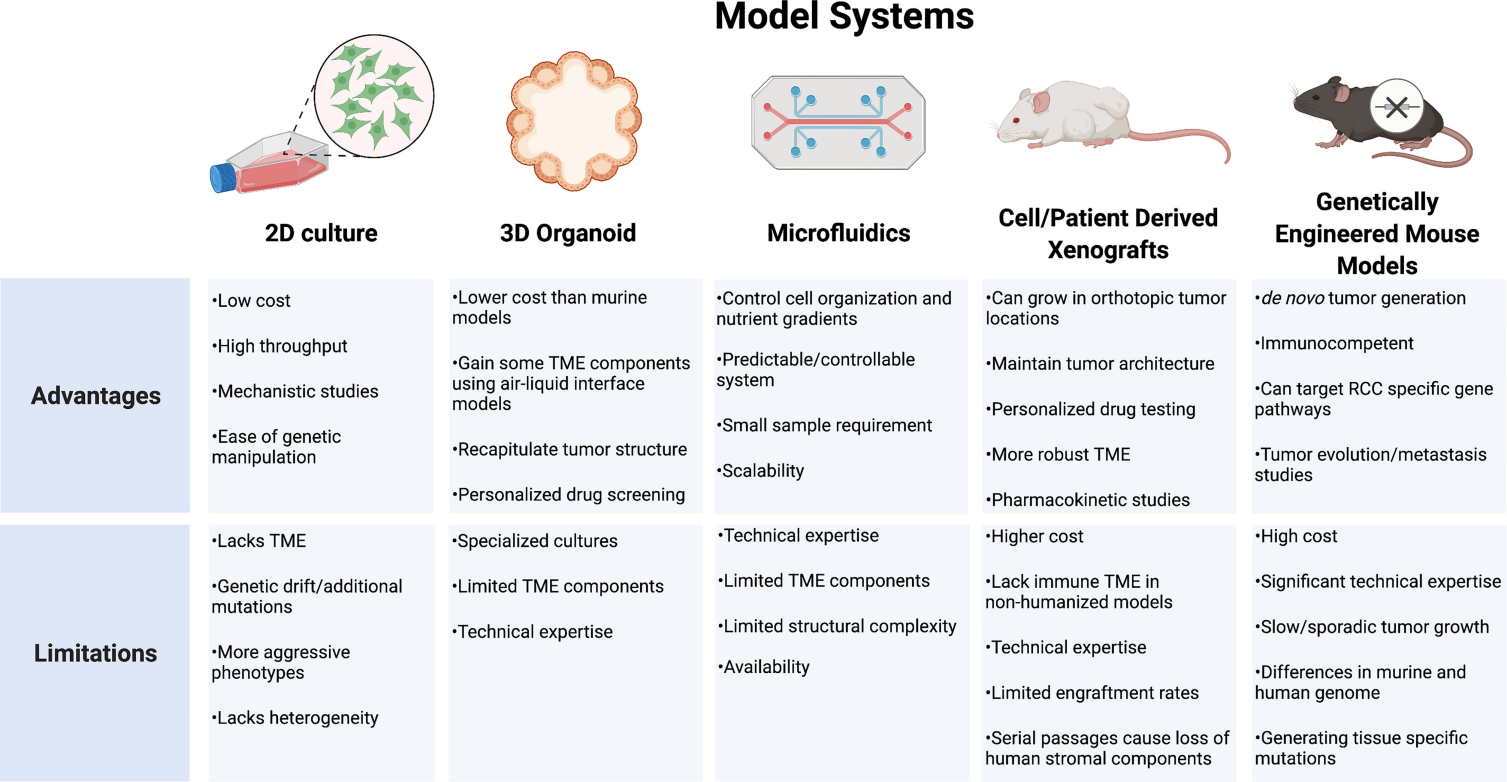
Model systems used for renal cell carcinoma research. TME, tumor microenvironment; RCC, renal cell carcinoma.

RCC models that incorporate a reliable representation of tumor genomic and phenotypic behavior in addition to the surrounding TME and host response are lacking. Strengths of the individual systems must be balanced against each system’s unique limitations (**Figure 4**). While cell lines have been the backbone of oncology research in understanding cellular mechanisms, these systems are limited by divergent genetic changes *in vitro* compared to human tumors. RCC cell lines frequently respond differently to drug therapy and lack a surrounding cellular microenvironment, which is important for RCC tumor biology. Organoids provide a more realistic tumor physical structure and may be developed with components of the surrounding TME, but still lack many of the complex components and interactions of the tumor environment. PDX models provide a more biologically relevant model which may be particularly useful to test drug therapies on individual tumors for a personalized medicine approach. These models, however, often require an immunocompromised mouse which limits the ability to study therapeutics targeting the immune response. Development of humanized models continues to expand in an effort to try and overcome these limitations. GEMMs provide an opportunity for the study of *de novo* tumor development and investigations into clonal evolution and metastases. These models feature immunocompetent hosts providing a more relevant platform for immunotherapeutic testing. All models require specific expertise and experience to effectively utilize. As each model continues to develop, further discoveries into the driver events of RCC will create new priorities for model development. The insights gained will allow for new diagnostic and therapeutic approaches as well as individualized approaches to RCC management.

## Author Contributions

All authors contributed to the conceptualization, writing, editing, figure and table development. All authors contributed to the article and approved the submitted version.

## Conflict of Interest

DB holds equity in Bellbrook Labs LLC, Tasso Inc., Turba LLC, Salus Discovery LLC, Stacks to the Future LLC, Lynx Biosciences Inc., Flambeau Diagnostics, and Onexio Biosystems.

The remaining authors declare that the research was conducted in the absence of any commercial or financial relationships that could be construed as a potential conflict of interest.

## Publisher’s Note

All claims expressed in this article are solely those of the authors and do not necessarily represent those of their affiliated organizations, or those of the publisher, the editors and the reviewers. Any product that may be evaluated in this article, or claim that may be made by its manufacturer, is not guaranteed or endorsed by the publisher.
